# Interstitial lung abnormalities: new insights between theory and clinical practice

**DOI:** 10.1186/s13244-021-01141-z

**Published:** 2022-01-15

**Authors:** Roberta Eufrasia Ledda, Gianluca Milanese, Francesca Milone, Ludovica Leo, Maurizio Balbi, Mario Silva, Nicola Sverzellati

**Affiliations:** 1 Unit of “Scienze Radiologiche” (Pad. Barbieri), Department of Medicine and Surgery (DiMeC), University Hospital of Parma, via Gramsci 14, 43126 Parma, Italy; 2 Department of Medicine and Surgery (DiMeC), University of Parma, Parma, Italy

**Keywords:** Interstitial lung abnormalities, Interstitial lung disease, Multidetector computed tomography, Terminology, Multidisciplinary approach

## Abstract

Interstitial lung abnormalities (ILAs) represent radiologic abnormalities incidentally detected on chest computed tomography (CT) examination, potentially related to interstitial lung diseases (ILD). Numerous studies have demonstrated that ILAs are associated with increased risk of progression toward pulmonary fibrosis and mortality. Some radiological patterns have been proven to be at a higher risk of progression. In this setting, the role of radiologists in reporting these interstitial abnormalities is critical. This review aims to discuss the most recent advancements in understanding this radiological entity and the open issues that still prevent the translation from theory to practice, emphasizing the importance of ILA recognition and adequately reporting in clinical practice.

## Keypoints

- Interstitial lung abnormality (ILA), perceived as a *niche* topic, is underreported in clinical practice.
- Adequate terminology is key for the adequate management of ILA.
- The role of radiologist goes beyond the mere recognition of ILA.

## Background

The use of chest high-resolution computed tomography (HRCT) in clinical practice and its continuous implementation have revolutionized the diagnosis of interstitial lung diseases (ILDs), contributing to an increasing interest in such topic by both radiologists and clinicians [1–3]. The interpretation of chest HRCT, however, is a complex process [2, 4] and the recognition of mild interstitial abnormalities might be rather challenging. In fact, the relative ease of assessing severe and extensively distributed abnormalities strongly contrasts with the difficulty of depicting subtle and less extensive ILD, at risk of being overlooked. Subclinical high density interstitial abnormalities can be displayed in patients undergoing either partial or complete chest CT examination, without clinical suspicion of underlying ILD. These incidentally detected CT findings, potentially representing symptomatic ILD, are now called interstitial lung abnormalities (ILAs) [5].

The reported association between ILA and adverse outcomes (e.g., all-cause mortality, hospitalization, progressive functional decline, increased lung cancer risk, etc.), as well as the blurred burden between ILAs and idiopathic pulmonary fibrosis (IPF) in some individuals, strongly suggest a potential clinical significance of ILAs. Although the progression of ILA toward pulmonary fibrosis has been proven in some patients ‘cohorts [6], not all cases of ILA represent or evolve toward overt ILD [7]. Current diagnostic strategies are focused onto the separation between clinically irrelevant ILA and clinically significant ILA, which might benefit from early treatment with antifibrotic therapies [5].

To date, the estimated prevalence of ILA in subjects older than 60 years of age is up to 9% in smokers and 7% in non-smokers [8], reaching the rate of 9.7% [9] and 25% [10] in lung cancer screening cohorts. Radiologists represent the frontline of ILA detection and characterization, somewhat of a gatekeeper in the gaze between early detection and/or overdiagnosis/overinvestigation. Because ILA is usually incidental, this (pre)clinical matter is extended beyond the cohort of chest radiologists. ILA, however, seems to be underappreciated by radiologists despite the incremental number of CT referrals for diagnostic purposes as well as the implementation of lung cancer screening programs [10–13].

Since ILAs were first described in tobacco smokers [14–16], numerous studies have attempted to characterize these CT abnormalities [6, 12, 17], leaving unanswered questions. Aiming at addressing some of these questions, the Fleischer Society has recently released a Position Paper that provides clarity to the definition, terminology, risk factors and management of ILA [5]. This review article discusses the new insight into this subclinical entity and the open issues that still prevent ILA to be appropriately managed. Future perspectives on a multidisciplinary approach of ILA and the potential role of quantitative imaging in such setting are also discussed.

## What’s new?

Prior to the Fleischner Society’s Position Paper, an heterogeneous terminology had been used to describe ILA, including (1) ILD at an early stage, (2) early ILD, (3) preclinical ILD and (4) subclinical ILD [5, 18, 19], all suggesting a relentless progression toward ILD. Such heterogeneity along with the lack of a standardized terminology has likely contributed to ILA been erroneously perceived as a *niche* topic, prerogative of either respiratory physicians or chest radiologists. The Fleischner Society’s Position Paper attempts to fill this gap, emphasizing the importance of ILA being appropriately recognized and reported by radiologists in their routine practice.

**Fig. 1:**
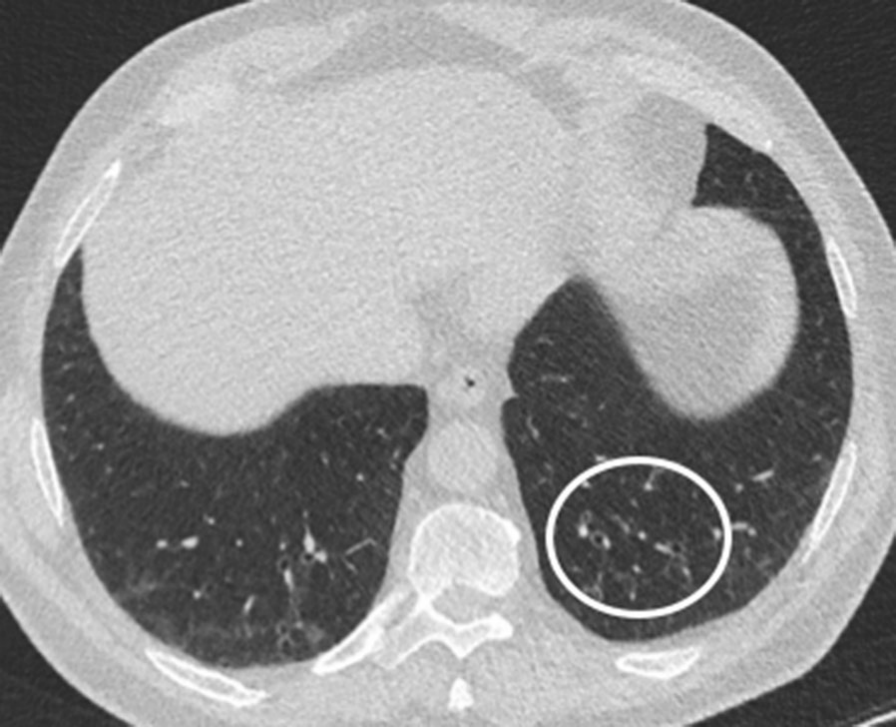
Axial CT image without contrast (slice thickness of 1.5 mm) of a 64-year-old lady shows bilateral GGOs in the lower lobes and fine reticular opacities (*white circle*) with subpleural sparing, compatible with non-subpleural ILAs

**Fig. 2:**
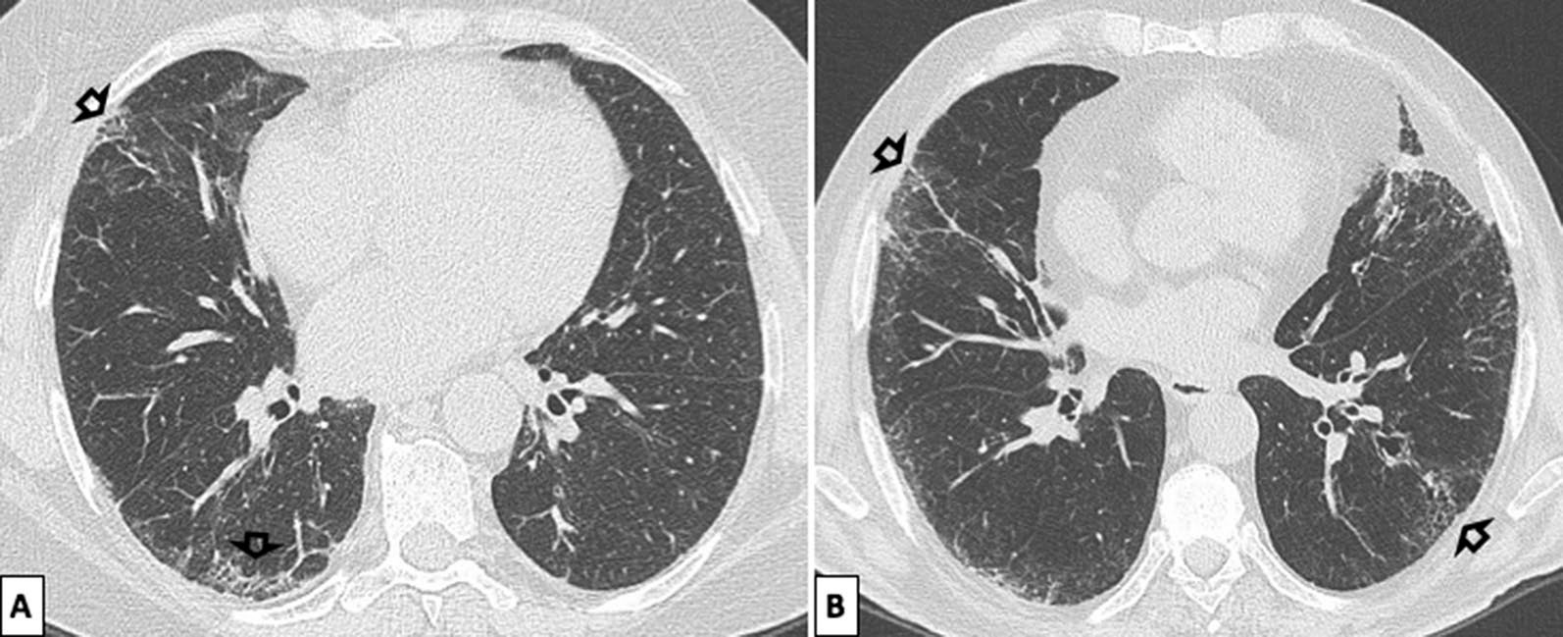
**a**, **b** Axial CT images without contrast (slice thickness of 1 mm) of a 77-year-old gentleman show bilateral GGOs and reticular opacities (*open arrows*) involving the subpleural regions of both upper and lower lobes. This pattern is compatible with non-fibrotic subpleural ILAs, since no overt signs of fibrosis, such as bronchiectasis or honeycombing, are displayed

**Fig. 3:**
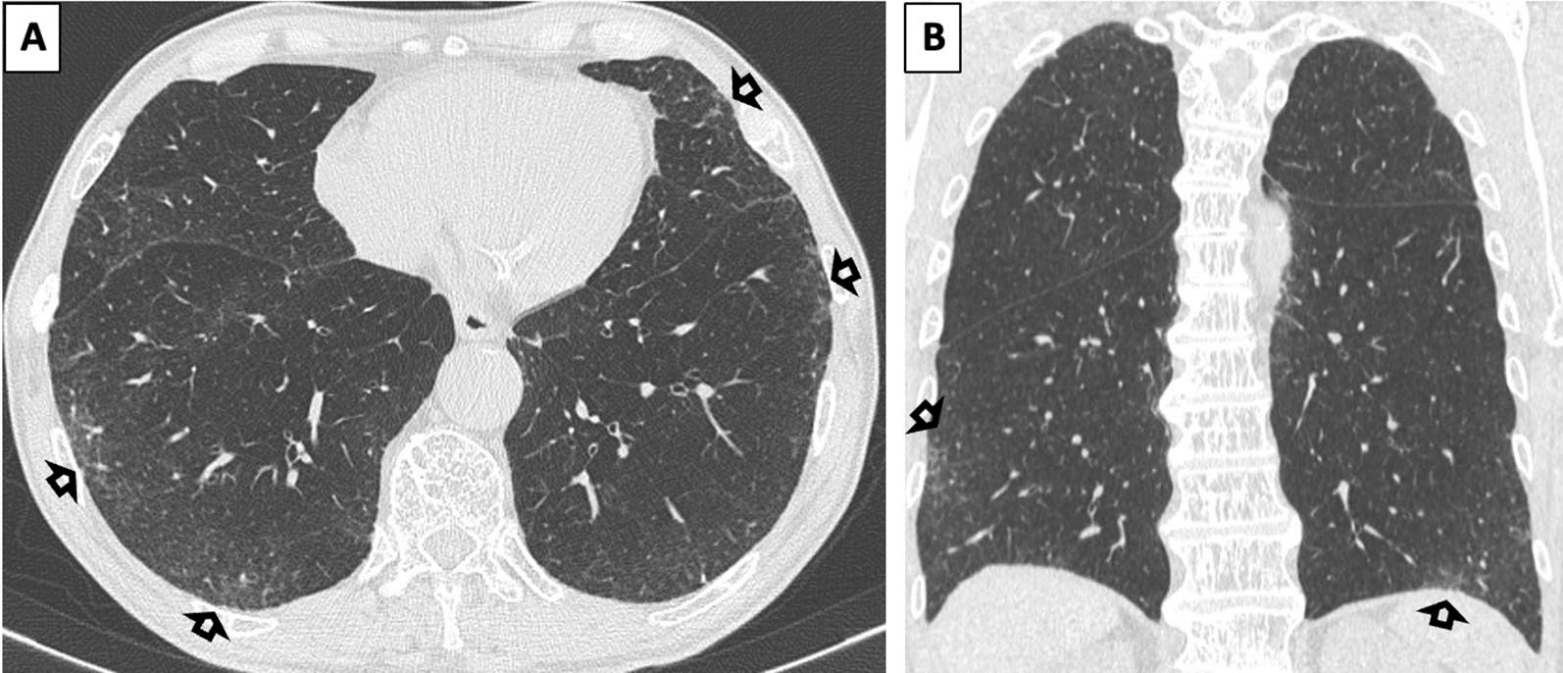
**a**, **b** Axial (**a**) and coronal (**b**) CT images without contrast (slice thickness of 1 mm) of a 79-year-old gentleman display bilateral GGOs and reticular opacities (*open arrows*) in the subpleural regions of both upper and lower lobes, compatible with non-fibrotic subpleural ILAs

**Fig. 4:**
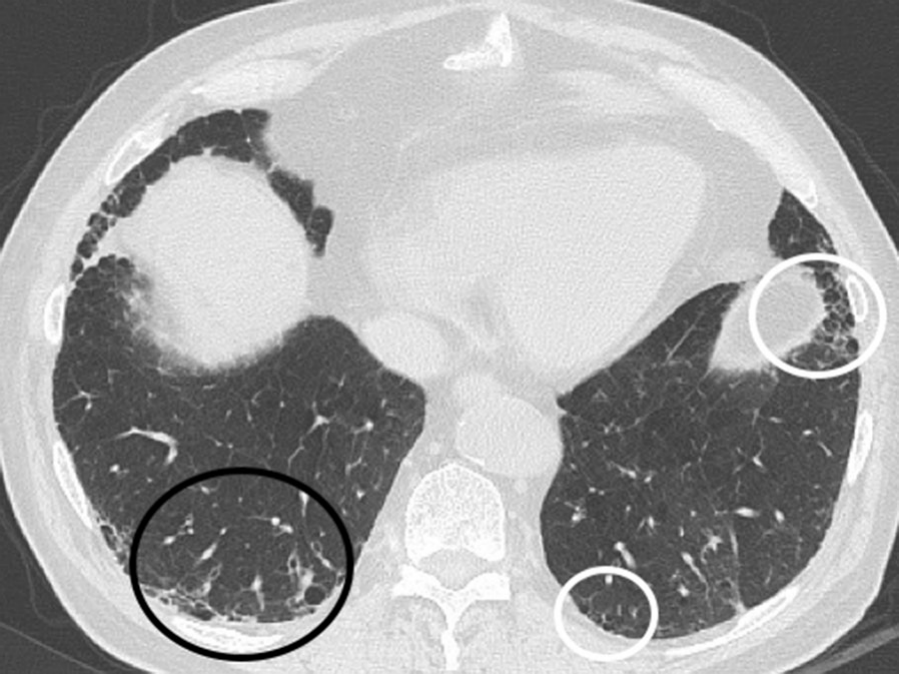
Axial CT image without contrast (slice thickness of 1 mm) of a 68-year-old gentleman shows traction bronchiectases (*white circle*) and some cystic airspaces with thick fibrous walls (*black circles*), which represent fibrotic parenchymal changes

**Fig. 5:**
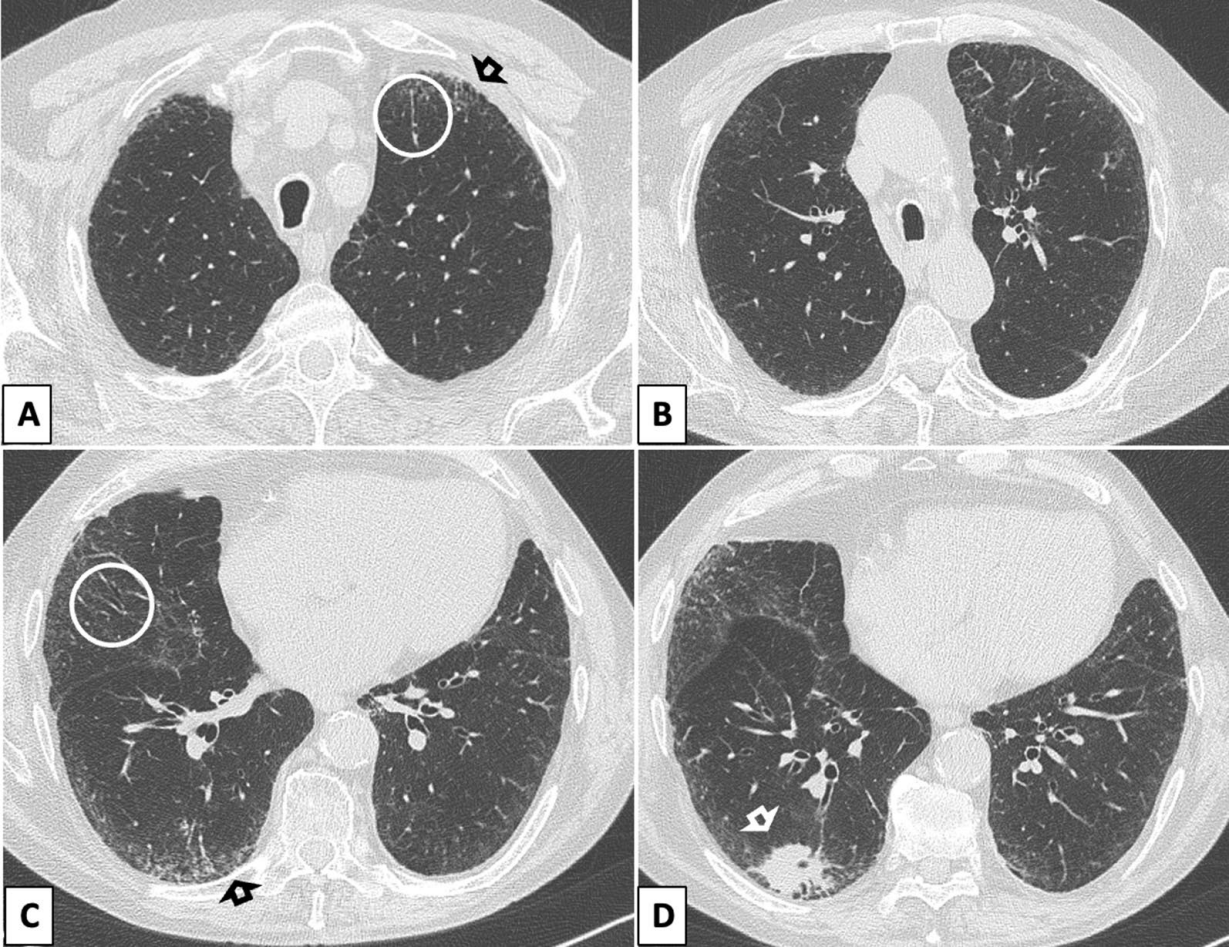
**a–d** Axial CT images (**a**–**d**) without contrast (slice thickness of 1 mm) of an 81-year-old gentleman show traction bronchiectases (*white circles*) and subtle cystic airspaces (*black arrows*), compatible with subpleural fibrotic ILA. (**d**) The CT scan, performed following the depiction of a nodular consolidation in the right lower zone at chest radiography, also showed a mass in the right lower lobe (white arrow) highly suspicious for primary lung cancer

**Fig. 6:**
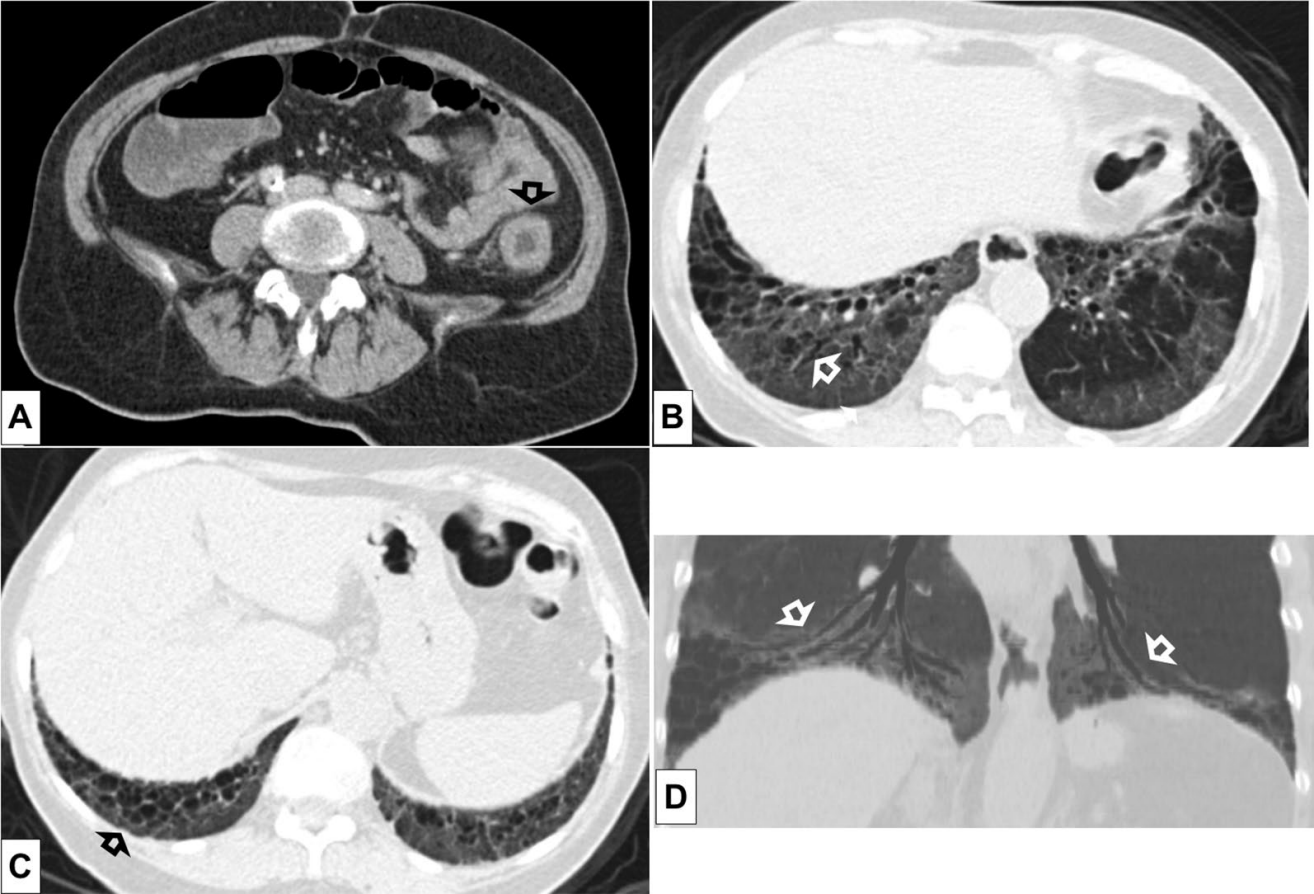
**a–d**. Axial (**a**) CT image of the abdomen with contrast (venous phase) of a 72-year-old gentleman shows wall thickening of the descending colon (*black open arrow*). Parenchymal window reconstructed axial CT images (**b**, **c** slice thickness of 2 mm) of the abdomen show right lower lobe traction bronchiectasis (*white open arrow*) and cystic airspaces with thick fibrous walls, suggestive of honeycombing (*black open arrow*). Minimum intensity projection (MinIP) reconstruction coronal image (**d**) shows lower-lobes traction bronchiectasis (*white open arrows*)

ILAs are defined as non-dependent lung abnormalities [20], diffuse in distribution (i.e., non-focal) and with at least 5% extent of a lung zone (upper, middle, and lower lung zones are demarcated by the levels of the inferior aortic arch and right inferior pulmonary vein), depicted in individuals in whom ILD is not suspected and thus, incidentally [5]. CT findings accounting for ILA include ground-glass or reticular opacities, honeycombing, and non-emphysematous cysts. Once such abnormalities have been recognized, radiologists are to report which lung regions are involved as different distribution patterns may have different prognostic implications [21]. In particular, Hatabu et al. proposed three CT categories of ILAs: (1) non-subpleural ILAs (Fig. 1), (2) subpleural non-fibrotic ILAs (Figs. 2, 3) and (3) subpleural fibrotic ILAs (Figs. 4, 5, 6), where CT features of lung fibrosis include architectural distortion (e.g., fissures displacement, bronchovascular structures distortion) with traction bronchiectasis, and/or honeycombing (or both) [5]. These categories were shown to be associated with different risk of progression and mortality [7, 9]. For instance, non-subpleural ILAs tend to remain stable and do not correlate with increased mortality, while the opposite is true for subpleural fibrotic ILAs [7, 9]. The distinction between fibrotic and non-fibrotic ILA is paramount yet challenging in limited or early disease (e.g., mild subpleural reticular opacities) because CT histologic correlation in this setting is imperfect. In one patient, limited subpleural reticulation may be the sole CT manifestation of histologically advanced fibrosis but in another may not represent fibrosis at all [22].

The exclusion of the centrilobular nodules from the list of ILAs features represents a meaningful change from prior definitions and remarks the importance of focusing on those CT abnormalities that are more at risk of progression [9]. Other CT findings that have been removed from the list of interstitial abnormalities potentially regarded as ILAs include focal and unilateral ground glass opacities (GGO) since they are unlikely to represent ILD (typically bilateral), patchy GGO and tree in bud nodularity, especially if involving the lower lobes as more likely related to aspiration [23].

Although the management of subjects with ILAs should be driven by both CT and clinical features, even fully asymptomatic individuals with ILAs can be subcategorized at risk of progression or not at risk of progression merely according to the CT pattern, here the pivotal role of radiologist in skilled triggering of work-up. Notably, subpleural fibrotic ILAs are deemed to be more at risk to progress toward frank pulmonary fibrosis. Putman et al. observed that evidence of subpleural reticular abnormalities, lower lobe changes and traction bronchiectasis were associated with a sixfold increase odds of radiological progression. They also reported that all cases displaying honeycombing had progressed over the following 5 years [7].

ILA is incidentally detected, but respiratory symptoms may be present, in which case such interstitial abnormalities likely represent ILD and are only temporarily labeled as ILAs. A set of clinical questions are suggested to rule out any respiratory symptoms that would require referral to pulmonologist otherwise. Critical clinical information includes history of cigarette smoking, other inhalational exposures, medications, radiation therapy and thoracic surgery [24].

The Fleischner Society’s Position Paper also emphasizes the importance of reporting the ILAs in cancer patients. The combination of cancer and ILA increases the risk of developing severe pneumonitis as a side effect of immunotherapy (immune checkpoint inhibitors), systemic chemotherapy and radiation therapy [25–29]. Furthermore, these subjects are more likely to suffer from surgery complications, including acute respiratory distress syndrome (ARDS) [30], suggesting that ILA might have clinical implication that goes beyond the risk of progression toward pulmonary fibrosis, and thus, reinforcing the importance of not to disregard such interstitial abnormalities.

## Open issues

Despite the remarkable contribution provided by Fleischner Society’s Position Paper [5], several questions still need to be addressed, some of which are related to limitations that might be appreciated in clinical practice at the time of both ILA first identification and subsequent evaluations.

### Identification of ILA

ILAs can be found on either abdominal or chest CT performed for any indication (Figs. 5, 6). This would trigger a chest CT to confirm and better characterize the CT abnormalities that were incidentally detected. However, in whom and when a dedicated CT should be performed is still an open question that warrants clearer recommendations. Furthermore, it might be questioned whether referring asymptomatic subjects for an additional CT scan is appropriate, particularly younger individuals because of the risks related to radiation exposure. The use of a low-dose chest CT protocol is accepted for evaluating fibrotic lung disease [31]. Nonetheless, more data are needed to understand the technical CT requirements for scanning subjects with suspected ILAs.

A chest CT scan optimized for the detection of interstitial abnormalities can be of value in symptomatic patients to provide detailed information on both findings and distribution, since different patterns of ILAs have been proved to have different implications in terms of risk of progression toward pulmonary fibrosis [7, 9]. Identifying symptomatic subjects who might benefit from an additional scan, however, can be challenging in a busy radiological department. Incidental findings, including ILAs, are usually depicted at the time of reporting, when patients are unavailable to be questioned. Moreover, investigating the presence of symptoms unrelated to the clinical indication for which the patient is referred, is rather difficult. In this scenario, standardized reporting is expected to sync radiological impression and the subsequent clinical management by referring physician, when appropriate. A pragmatic approach is offered by the National Health Service (NHS) of England in their document for targeted screening of lung cancer by low-dose CT [32], but dedicated analyses are still eagerly awaited. In case of mild ILD, symptoms can be subtle, and their identification might require a set of detailed questions. Furthermore, recognizing subjects at high risk of progression among those with no symptoms appears not less challenging, since investigating the presence of both clinical and demographic risk factors can be likewise problematic.

It must be acknowledged that most data on ILAs were obtained from studies involving research participants from general population cohorts, and from populations of smokers enriched for the presence of chronic-obstructive pulmonary disease (COPD), cardiovascular and lung cancer screening cohorts [9, 11, 12, 24, 33–37]. Indeed, those cohorts mostly included either elderly [9, 38, 39] or smokers [14, 34], thus subjects at greater risk of ILAs. Although incidental findings have also being increasingly recognized in every type of CT scan, further data are needed to figure out the prevalence of the ILAs in daily practice.

### Progression of ILA

Disease progression at CT is of both diagnostic and prognostic value. However, definition of disease progression deserves further clarification. In fact, ILAs as well as any ILD may change in both extent and morphology over time. However, it is not clear how disease progression should be assessed. ILAs are often mild in extent and a marginal longitudinal increase may be difficult to appreciate. Therefore, an accurate anatomical matching of the CT images obtained at different timepoints is at least required to evaluate the longitudinal behavior of the ILAs. Furthermore, it is not clear if and to what extent the development of new findings (e.g., new traction bronchiolectasis, more reticular opacities, etc.) should be regarded as established signs of disease progression.

**Fig. 7:**
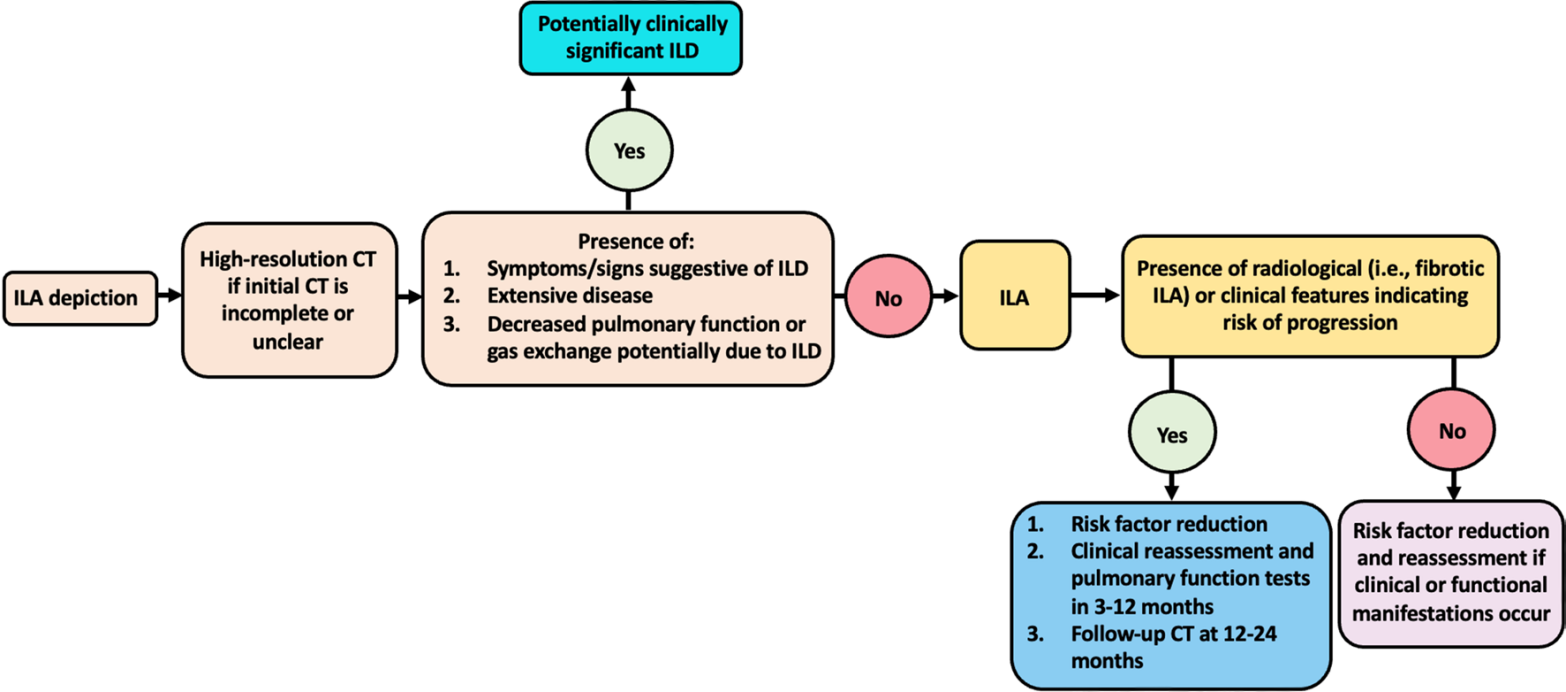
Flowchart summarizing the suggested management of ILA. Adapted from Reference [5]

Whether a mere radiological progression should be managed as a progressed ILAs regardless of the presence of respiratory symptoms is still to be defined. Araki et al. demonstrated a correlation between radiological and functional progression, observing that patients with evidence of radiological progression showed an accelerated decline in pulmonary function [6]. However, evidence of radiological progression might not necessarily be associated with a worsening of pulmonary function and/or symptom occurrence [40]. Should these cases be regarded as progressed? Targeting high-risk patients is crucial from a therapeutic perspective, since the use of antifibrotic drugs, already employed in IPF [31, 41], has been proposed in ILAs patients at high risk of progression. The definition of specific risk factors for progression will also allow to follow-up these subjects appropriately. Up to now, the correct clinical and radiological follow-up timing is still unknown. A clinical 3–12 months follow-up along with a repeat CT scan at 12–24 months is deemed acceptable for ILAs patients at high risk of progression (Fig. 7), in whom a further follow-up after the first year can be considered [5]. Follow-up frequency and duration after the first year has not yet been defined and the optimal method for assessment of progression of such minor findings is still to be analyzed, as previously done in clinically apparent ILDs [42, 43].

Interestingly, Copley et al. observed ILAs in normal subjects over the age of 75 years and not in subjects with less than 55 years of age [44]. They also reported that the majority of these abnormalities were not associated with respiratory symptoms nor with declined pulmonary function and therefore, not in need of follow-up or treatment. However, the borderlands of the normal for the ILAs are still not defined [45].

## Future perspectives

As discussed above, mild interstitial abnormalities might have already been causing symptoms and some are at high risk of progression [46]. A correct management demands a thorough patient assessment, which should spark from skilled radiological characterization. However, any general or subspecialty radiologist might encounter these findings and is expected to play a role in their management, mostly guided by the risk of progression and thus, by the CT pattern, either fibrotic or non-fibrotic, whose definition is entirely on radiologist. Nonetheless, beside the characterization of the CT pattern, radiologist can provide practical recommendations, suggesting a radiological follow-up for fibrotic ILA or a pulmonary evaluation for symptomatic subjects. In this context, a multidisciplinary approach to this radiological entity seems to be the optimal management and thus, should be encouraged. The risk stratification by a comprehensive evaluation of predisposing factors might be of great value for focusing on appropriate elevation of ILA into the clinical domain, eventually helped by investigation of symptoms. This approach might be easier and more relevant in patients with other comorbidities (e.g., COPD). Noteworthy, patients with history of ILA and COPD are at a higher risk of pulmonary fibrosis and COPD exacerbations [33].

Establishing the extent of ILAs by a purely visual assessment may not be an easy task for radiologists, even for chest radiologists among whom there is a high interobserver variability. The identification of ILA and its pattern recognition is indeed subjective to reader expertise and substantially affected by interobserver variability, even among experts [22, 47–49]. Notably, Walsh et al. demonstrated that even in case of overt lung fibrosis, the interobserver agreement among thoracic radiologists of varying levels of experience is at best only moderate [50].

The 5% threshold embraced by the Position Paper to define the presence of ILA seems quite prone to subjective interpretation, while the accurate quantification of the abnormal parenchyma is of utmost importance at the time of both diagnosis and follow-up. Signs of frank progression can be easily detected as compared to subtle changes, which are more likely to be overlooked. However, subtle progression of minor findings might still represent a substantial relative increase in extent (progression from 6 to 8% extent represents a relative increase in one third).

Quantitative imaging techniques, successfully used to score the proportion of parenchymal involvement in diffuse lung diseases [51–55], ensure objectiveness and reproducibility and might be efficiently employed also in ILA. However, significant discrepancies between visual and automated assessment in ILD have been described and future studies will be needed to overcome this limitation. Jacob et al., for instance, observed how fine reticular abnormalities overlaying on GGOs were scored as merely GGOs by the quantitative software [42]. Of note, when fibrotic changes (more often involving the lower lobes) progress, the healthy lung regions (usually the upper lobes) might hyper-expand, as a result of a compensatory response [56]. These changes can be misinterpreted by the quantitative software, for which an increased extent of fibrosis in absolute terms may be scored as a relatively smaller volume of fibrotic lung and thus, underestimated [42]. Recent advances in the field of artificial intelligence and machine learning seem to offer great opportunities in medical imaging [57, 58]. A detailed discussion of the numerous potential applications of machine learning in diagnostic imaging goes beyond the aim of this review. Among others, such applications include the possibility for integrating radiological and clinical data, which will help in redefining the real risk of progression at the time of diagnosis and establishing the severity of progression.

## Conclusion

Despite the remarkable advances in understanding this radiological entity, several issues are still debated. Undoubtedly, the Position Paper recently released by the Fleischner Society has addressed some of the open questions, giving a major contribution on definition and terminology, easing the important task of reporting these often subtle interstitial abnormalities. Future studies on general population, however, are fostered to investigate ILAs risk factors, whereas a multidisciplinary approach seems to be the right answer to the optimal management.

## Data Availability

Not applicable.

## Abbreviations

ARDS: Acute respiratory distress syndrome
COPD: Chronic obstructive pulmonary disease
CT: Computed tomography
GGO: Ground glass opacity
HRCT: High resolution computed tomography
ILAs: Interstitial lung abnormalities
ILD: Interstitial lung disease
IPF: Idiopathic pulmonary fibrosis
NHS: National Health Service
